# Genetic diversity and relatedness amongst captive saker falcons (*Falcocherrug*) in the Green Balkans’ Wildlife Rehabilitation and Breeding Centre in Bulgaria

**DOI:** 10.3897/BDJ.11.e105863

**Published:** 2023-07-13

**Authors:** Rusko Petrov, Thierry Hoareau, Loic Lesobre, Yana Andonova, Dobry Yarkov, Nayden Chakarov

**Affiliations:** 1 Green Balkans - Stara Zagora NGo, Stara Zagora, Bulgaria Green Balkans - Stara Zagora NGo Stara Zagora Bulgaria; 2 Trakia University, Stara Zagora, Bulgaria Trakia University Stara Zagora Bulgaria; 3 Reneco International Wildlife Consultants LLC, Abu Dhabi, United Arab Emirates Reneco International Wildlife Consultants LLC Abu Dhabi United Arab Emirates; 4 Green Balkans - Stara Zagora NGO, Stara Zagora, Bulgaria Green Balkans - Stara Zagora NGO Stara Zagora Bulgaria; 5 University of Bielefeld, Bielefeld, Germany University of Bielefeld Bielefeld Germany

**Keywords:** genomics, birds of prey, saker falcon, captive breeding, re-introduction, biodiversity

## Abstract

The globally endangered saker falcon (*Falcocherrug*) is currently being re-introduced in Bulgaria, where the falcons are bred in captivity and released through the hacking method. We relied on the birds’ pedigree when forming the breeding pairs from 2011. In 2021-2022, we had the opportunity to evaluate our captive population via DNA tests. We performed the first genetic assessment of the sakers in the WRBC through a genome evaluation of the most important founders (n = 12) and, in 2022, we executed a microsatellite analysis on 30 saker falcons from the programme. We compared the results with the known pedigree and history of the saker falcons. The genetic tests helped to assign relatedness to some birds with missing or incomplete pedigrees, indicating the test can complement that information and lead to better management of the captive group. One pair was separated as a precaution as it was indicated by one the tests that the two birds are more closely related than expected. The research could be beneficial to other raptor captive breeding programmes dealing with a similar group composition.

## Introduction

The saker falcon (*Falcocherrug*) is a globally endangered species by the categorisation of the International Union for Conservation of Nature (BirdLife International 2021). In Bulgaria, at the south-eastern edge of its range in Europe, the saker falcon is marked as critically endangered in the Red Data Book of the Republic of Bulgaria (Domuschiev et al. 2015). Currently, the only known successfully breeding pairs of saker falcons in the country are formed of re-introduced birds, part of the ongoing programme to restore the local breeding population. Since 2011, there has been a captive breeding group performed by the Wildlife Rehabilitation and Breeding Centre - Green Balkans (WRBC), part of Green Balkans - Stara Zagora NGO. These sakers’ offspring are being released through the hacking method (Sherrod et al. 1987) in the Upper Thracian Plain in Bulgaria. The objective of the current stage of the species restoration project is to release 100 juvenile sakers over a five-year period (2020-2024). They are retained in the hack sites until independence through food provision in order to imprint on the area and return to it when they reach breeding age at around 3-years-old (Dixon et al. 2020, Lazarova et al. 2021, Petrov et al. 2021, Petrov et al. 2022).

Captive breeding with the objective of population restocking or re-introduction is increasingly relied upon to prevent the local or global extinction of species (Conde et al. 2011, Pritchard et al. 2011, Ralls and Ballou 2013, McGowan et al. 2016). When breeding animals in captivity, importance should be placed on the genetic relatedness and diversity of the group, especially when the aim is re-introduction. In order for rearing healthy offspring which will be able to form a future self-sustaining population, the founding individuals should be carefully chosen and paired. There are certain risks associated with maintaining a captive breeding group of animals - too-small gene pool can lead to reduced genetic diversity in the long run and increased prevalence of genetic disorders and inbreeding depression (IUCN/SSC 2013). Captive breeding programmes typically use pedigrees to manage genetic diversity and avoid inbreeding by analysis of stud books which contain data on births, deaths and parental information; however, they can often be incomplete and overestimated (Rabier et al. 2022). Managing the captive group can be a challenge if there are such deficiencies. DNA analysis can help fill in these gaps and aid the genetic management of the breeding stock (Allendorf et al. 2010, Frankham et al. 2010).

The aim of this research was to gain further knowledge of the individuals in the WRBC saker falcon captive breeding group, beyond the history of arrival and pedigree which were incomplete for some of the birds. In our saker falcon breed-and-release programme in Bulgaria, we relied on the birds’ pedigree when forming the breeding pairs from 2011. Recently, in 2021-2022, we had the opportunity to evaluate our captive population via DNA tests. We performed the first genetic assessment of the sakers in the WRBC through a genome evaluation of the most important founders (n = 12) and a microsatellite analysis on 30 saker falcons from the programme.

## Materials and Methods

### Breeding programme

Between 2011 and 2022, from the start of the captive breeding efforts to date, there have been, on average, 21 saker falcons in the WRBC captive breeding programme (range 4-29) (Table 1). There were, on average, 10 formed pairs each year (range 2-13), on average eight of them exhibited breeding behaviour (range 2-12), an average of five laid eggs in one or two clutches (range 0-12) and out of them, an average of four pairs per year reared chicks (range 0-8). The founding individuals were obtained from breeders from Central Europe and the UK.

### Samples

In 2021, a total of 12 samples, representing the most important founding individuals from the captive population of the WRBC with unclear background, were analysed through whole genome sequencing. Some of the saker falcons had been wild-caught and there were no data on them, others needed to be tested to clear relations in order to form breeding pairs of not closely-related birds. Seven of these 12 birds subsequently bred successfully (excluding Frodo, Arnold, Luna, Barir, Ariel). One individual - Bilbo, was known to be the offspring of Thomas and Lucia. Some of the other founders were not alive at the time of sampling and the other untested saker falcons were with known pedigree. Sample details of the 12 tested individuals are listed in Table 2.

In 2022, 30 samples of sakers from the breeding group were analysed at nine microsatellite loci - SSR11, SSR15, SSR45, SSR48, SSR53 SSR57, SSR63, SSR82 (Hou et al. 2018), Fp92 (Nesje et al. 2000). Details of these individuals are listed in alphabetical order in Table 3. Microsatellite genotyping was performed as described by Chakarov et al. (2013). M13-tagged primers for the corresponding loci were used in a 10 μl polymerase chain reaction (PCR) volume with 20–200 ng DNA, which was amplified for 35 cycles using a Type-it microsatellite PCR kit (QIAGEN), following the manufacturer's standard protocols and using an annealing temperature of 56°C. Diluted amplification products (1 μl; 1:20 dilution) were then resolved on an ABI 3730 Automated DNA Analyser (Applied Biosystems). Fragment lengths were scored for all individuals using Genemarker 1.95 (SoftGenetics LCC). Genotypes were analysed with Colony 2.0.6.6 (Wang 2013) with polygamy allowed for both sexes (option chosen given the possibility of re-pairing of individuals).

All birds were examined by a veterinary physician upon blood collection and were determined to be clinically healthy. Surfaces were disinfected with Desclean solution. We disinfected the area and collected 0.1 ml of whole blood from either left or right basilic vein (Vena cutanea ulnaris superficialis) of all specimens tested. We immediately placed the blood into Eppendorf collection tubes of 1.5 ml volume containing 1 ml 90% alcohol. We used 3 ml syringes with 23G needles.

### Genomic data

For the 12 founders, the original genomic data were first analysed to estimate genetic parameters (genomic coverage, number of informative variants, genetic diversity, relatedness and inbreeding coefficient) and were applied to a clustering analysis (principal component analysis: PCA).

The genomic data has been generated by the Beijing Genomic Institute (China) and included DNA extraction, library construction and whole genome resequencing at an expected coverage of 10×. As a reference for read mapping, we have used a chromosome-level genome assembly available for gyrfalcon on the NCBI portal (BioProject ID PRJNA561988; https://www.ncbi.nlm.nih.gov/assembly/GCF_015220075.1). The quality of this assembly is higher than the reference saker genome assembly. Moreover, gyrfalcons and sakers are closely-related species that are sometimes difficult to genetically differentiate - a recent study has shown that saker and gyrfalcon have the same genome organisation (Joseph et al. 2018).

### Read mapping, SNP calling and filtering

The quality of the Illumina paired-end reads (~ 21.7 GB of FASTQ files per falcon specimen) were first checked using the programme FASTP (v.0.20.0; https://github.com/opengene/fastp). These reads were then mapped on the gyrfalcon reference genome (1.12 GB of FASTA file) using BWA-MEM (Li and Durbin 2009). The obtained SAM files were then compressed and merged into a single BAM file (~ 21.7 GB per individual), which was then sorted and indexed using SAMTOOLS (Li et al. 2009). Duplicates were marked and removed from the BAM files using the PICARD tool. Variant sites2 were then identified using SAMTOOLS mpileup (Li et al. 2009) and called using BCFTOOLS (v.1.6.33) calls function (http://samtools.github.io/bcftools/bcftools.html). For the mapping, the identification and call of the variants were performed on each chromosome in parallel, which helped speed up the analyses. These analyses produced multiple VCF compressed files that contained all the information on the variant sites. To select high quality variants, we used functions available in VCFTOOLS and BCFTOOLS that include a selection of filters:

- Genotype phred-quality score of 30: Q30 indicates an error rate that represents one incorrect base call in 1000 times.
- a maximum of two alleles per single nucleotide polymorphism (hereafter SNP).
- a maximum of 50% missing data per site.

### Analysis of genomic diversity

The basic genomic statistics were obtained per individual using the whole genomic dataset. Three genetic parameters that include observed heterozygosity (Ho), inbreeding coefficient (F) and genetic relatedness (r) were calculated using BCFTOOLS (Danecek et al. 2011) from polymorphic biallelic variants. Finally, we used the plink v.1.90 package to generate a PCA. This programme starts by pruning variants to avoid linkage disequilibrium before performing the multidimensional analysis, which results in a lower number of variants suitable for the analysis.

## Results

### Reference genome

A single reference assembly was selected. It is the chromosome-level assembly obtained for gyrfalcon from the Vertebrate Genomes Project initiative (Bioproject PRJNA561988, release 03/11/2020; https://www.ncbi.nlm.nih.gov/assembly/GCF_015220075.1). It represents 25 haploid chromosomes and plasmids for a total sequence length of 1.2 Gbases and an average size of 49.8 million bp for the chromosomes, ranging from 126.9 to 0.4 million bp. The assembly counts a total of 108 scaffolds (unplaced sequences) that represent a total of 4.6 million bp.

### Read mapping and coverage

The whole genome sequencing for the 12 gyrfalcon samples produced a total of 260 GB of clean data (average of 21.7 GB per individual) that provided an average of 231 million reads per individual, ranging from 165 to 355 million reads per individuals (Table 4). The duplication rate is low with 1.0% on average, ranging from 0.3 to 1.7%.

**Figure 1.**
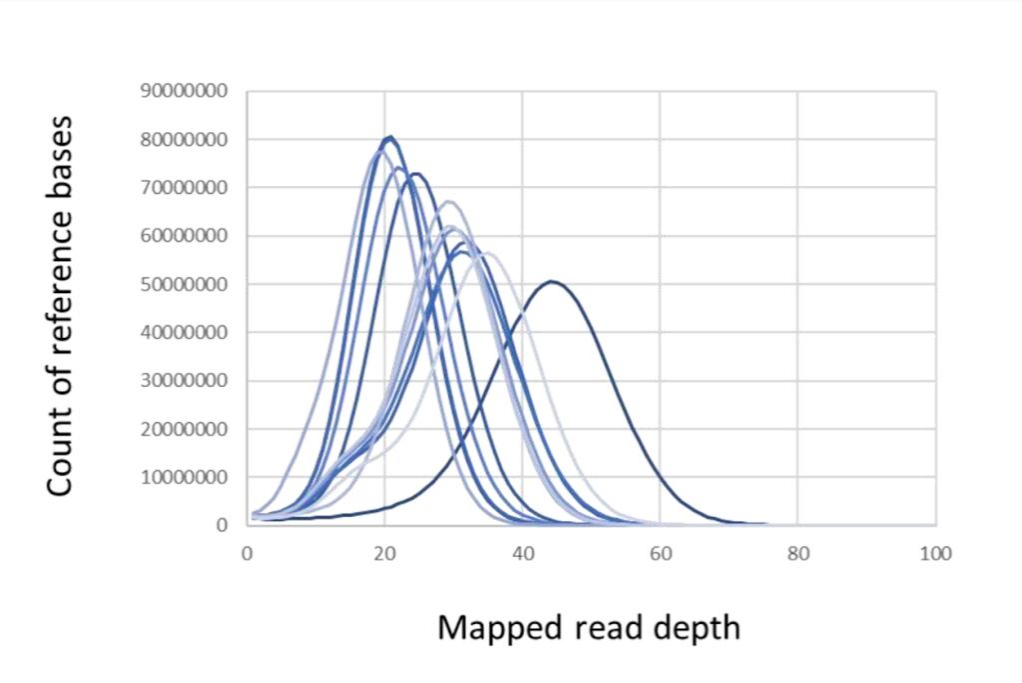
Distribution of genome sequence coverage for individuals of saker falcons from the captive population. The coverage is 28.1× on average across samples and ranges from 19× to 44×.

When considering the full dataset, the sequencing depth is around 28.1× coverage on average for each individual with 95% of the reference bases covered more than nine times in most cases (Table 5; Fig. 1).

### Genetic diversity and genetic relatedness amongst individuals

**Figure 2.**
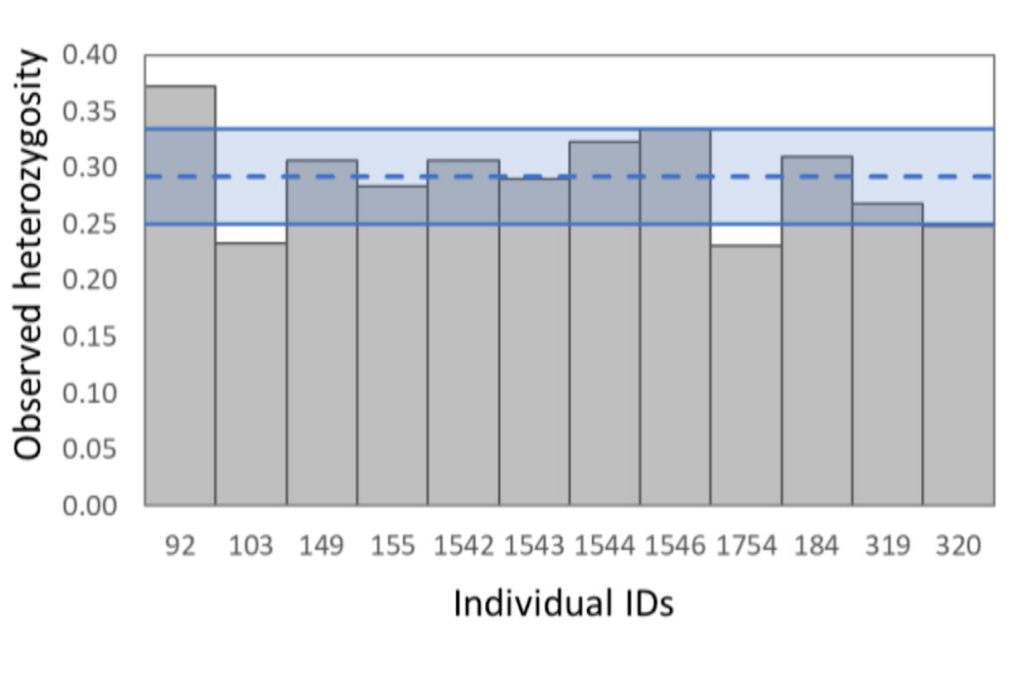
Genetic diversity of the different individuals as measured by the observed heterozygosity. The average observed heterozygosity (and standard deviation) calculated across samples (Ho = 0.293 ± 0.042) are represented by the horizontal lines and blue shading.

The overall genetic diversity of the samples are 21.89×10^-5^ ± 6.77×10^-5^ for nucleotide diversity (π) and 0.293 ± 0.042 for observed heterozygosity. Some individuals like 0103 and 1754 show lower genetic diversity while one, 092, show higher genetic diversity when compared to the rest of the samples (Fig. 2).

**Figure 3.**
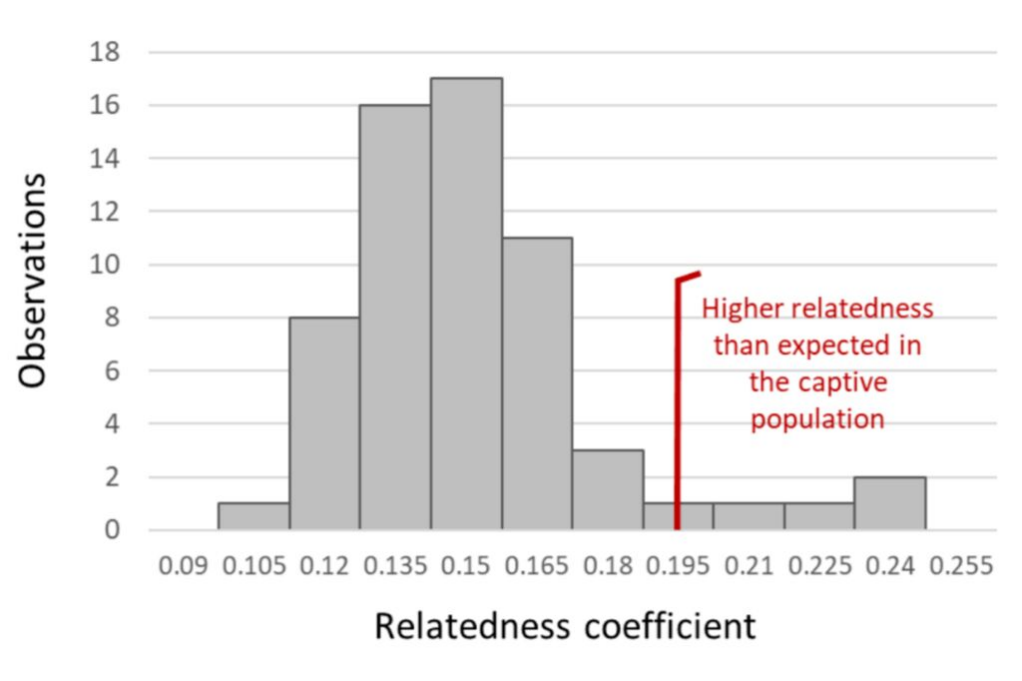
Frequency distribution of genetic relatedness performed on the 12 samples. Note that several comparisons of individual pairs show higher relatedness than expected amongst the founder individuals if they were unrelated. These pairs involve individuals 092, 1544 and 1546 and the individuals 1543, 184 and 319, all characterised by relatedness values around 0.2 and above.

The frequency distribution of the genetic relatedness coefficient (r) shows that some saker pairs were more highly related than the sample average, i.e. when individuals were unrelated (Fig. 3). The average value of the relatedness coefficient for all samples is 0.126 ± 0.089 and around 94% of the pairwise comparisons are below a relatedness value of 0.2. Individuals involved in these higher values of relatedness involve individual 092 that show a higher value of relatedness when compared to 1544 (r = 0.243) and 1546 (r = 0.239). The individual, 1543, also has high relatedness values when compared to individuals 0184 (r = 0.218) and 0319 (r = 0.205).

### PCA-based genetic clustering

**Figure 4.**
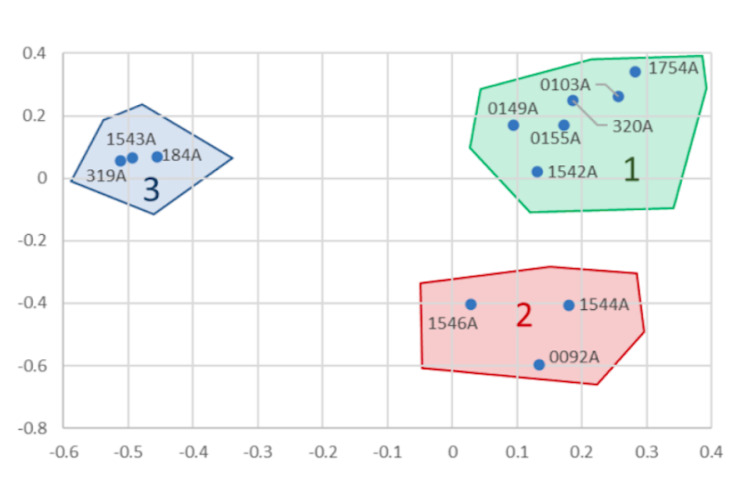
PCA illustrating the genetic difference amongst founding individuals of saker falcons from the captive population. Three clusters named 1, 2 and 3 can be observed and the colour shadings highlight these clusters.

Applying the PCA to the current variant dataset, several groupings can be observed (Fig. 4). On this figure, each point represents the genetic sample of an individual with its identifier and the distances between points are correlated with genetic distances. When individuals are close on the figure, this means that they are closer genetically than other individuals that are found more distant on the figure. There are three groups that can be observed. The most distinct is group 3 that comprises 0184, 0319 and 1543 and is separated from the two other groups along the first axis. Groups 1 and 2 are differentiated along the second axis. Group 2 is composed of parents and an offspring (092).

### Microsatellite analysis

The Colony analysis of microsatellite data arranged the sampled individuals as family/cluster members - the 30 birds were grouped into 17 clusters - 11 of which were represented by only one saker and the other six by two or more which are putatively related. These results are presented in Table 6.

## Discussion

### Quality of the genomic data

The blood tissues provided good quality of genomic data with high number of reads and low level of duplication. The sequencing depth ranges from 19× to 44× with 95% of the reference bases covered more than nine times.

### Genetic diversity and relatedness of founder individuals

The analyses of genetic diversity of the different individuals indicated that some individuals have lower genetic diversity than the rest of samples (Barir & Ariel), which may suggest a higher level of inbreeding. Moreover, one individual showed higher genetic diversity than the rest (Bilbo). The relatedness analyses indicated several individuals that seem to be more closely related than expected in the overall samples. These samples included the individual Eurydice that seems to be related to Frodo and Arnold and the individual Bilbo that is found to be kin related to both Thomas and Lucia.

### Genetic clustering amongst founder individuals

The search for genetic structure within the founder individuals of the saker population revealed genetic differences between individuals. It is unclear whether these differences underlie genetic differences between wild individuals or differences related to genetic drift associated with breeding strategies in the captive populations over multiple generations. It is important to note that two of the tree genetic clusters observed on the multidimensional analysis involved the individuals that are suspected to be kin related from the relatedness analysis. These results are in support of a genetic structure potentially arising from the breeding strategy rather than the underlying genetic structure in the wild.

### WRBC saker stud book

**Figure 5.**
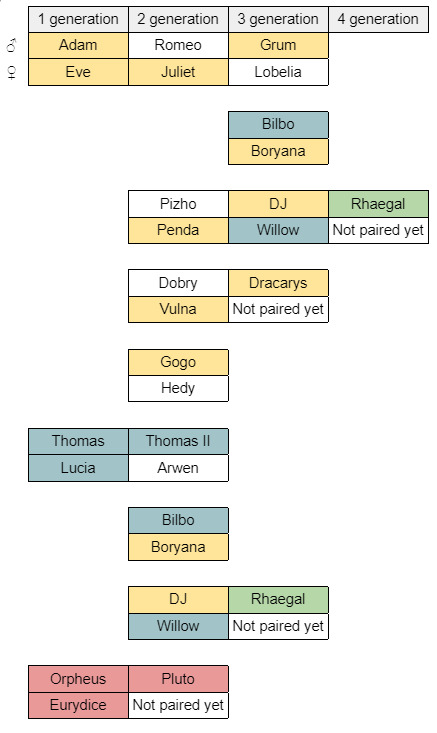
Family tree of the founding pairs and their unreleased offspring, left in the WRBC breeding programme.

Due to the lack of wild saker falcons in Bulgaria, birds from the western population of the species (*Falcocherrugcherrug*) were obtained from breeding sources in Central Europe and the UK. The saker falcon breeding group at the WRBC consisted of three founding pairs - Adam & Eve (A&E), Orpheus & Eurydice (O&E) and Thomas & Lucia (T&L) (Fig. 5). The offspring of A&E and T&L have formed the rest of the breeding pairs. Five of A&E’s offspring (Juliet, Penda, Plamena, Vulna, Gogo) have formed pairs with imported unrelated individuals. Two of T&L’s offspring (Bilbo and Willow) have formed pairs with A&E’s ‘grandchildren’ (Boryana and DJ). From the date when the saker falcons are paired in the WRBC, the family ties are recorded in a breed registry. However, not all prior relations were known, as it is often the case with pedigrees according to Rabier et al. (2022). Four new relations were uncovered through the tests, one of them required a pair to be separated as a precaution (T&L). Some of the findings were inconsistent between the two DNA tests made, so more research is required for more definitive results.

### Comparison between stud book and microsatellite data

There were a number of close-kin relations that were known from our pedigree data, but did not show up in the microsatellite analysis, indicating it could not replace the pedigree records, but can complement them. The test correctly determined certain male parent-offspring relationships (cluster 11: Pizho & DJ; cluster 7: Dobry & Dracarys) and certain sibling relationships (cluster 6: Bilbo, Willow & Thomas II; cluster 8: Vulna, Penda, Plamena & Gogo; cluster 12: Lobelia, Frodo & Arwen). However, Thomas and Lucis’s offspring were not in a cluster with any of them nor was Rhaegal - he was not in either DJ’s or Willow’s (his parents). Nevertheless, he also had a very low probability of not having close relatives in the sample.

Revelations stemming from the microsatellite results included three of the sakers - Shira, Arnold and Eurydice. Shira was confiscated in Bulgaria in 2020, far away from the single wild saker breeding territory known at the time - assumed to be an unrelated wild bird from a different line. She was included in the breeding programme. Being genetically associated with T&L’s progeny, it appears most likely that she was instead hatched in captivity in the Breeding Centre for Birds of Prey in Burgas, Bulgaria (breeding saker falcons for commercial purposes, where Thomas and Lucia resided that year), sold to a falconer, escaped and then taken in by a private home in 2020. By the same logic, Arnold may be an offspring of Adam and Eve, as are the others from cluster 8. It could have hatched before we obtained the pair. However this is not confirmed from the genomic study, placing it close to Frodo and Euridice. Eurydice came from the Czech Republic with incomplete pedigree, together with Lobelia, Frodo and Arwen. Both tests in this case clarified the origin of this individual - she seemed to descend from the same parents, however from an earlier clutch as she is older than the other three birds which were known to be from one clutch.

### Comparison between stud book and genomic data

Through the test, it was discovered that birds which were previously thought to be unrelated - Thomas, with a ring from Slovakia and Lucia, with a Czech ring, are closely related. They had been paired before that, in 2014, as 4-year-old birds and had proved to be a very successful pair, raising a total of 81 chicks since then. The results indicated that their offspring Bilbo has the highest genetic diversity of the other 12 tested falcons. Most of their other offspring were reared when the pair were in the Breeding Centre for Birds of Prey, so the chicks were sold for the purposes of falconry. However, following these results, as a precaution, from 2023, they will not form a breeding pair together in the WRBC so their progeny will not be released in the wild. The relatedness of T&L was not confirmed by the much sparser microsatellite data. The aim of breed-and-release programmes is to preserve the initial genetic diversity of the captive population (Ivy and Lacy 2012, Ralls and Ballou 2013). This is the reasonwhy, following the study, the offspring of A&E’s line will be crossed with ones from O&E’s line, with the aim to create additional breeding pairs which will produce healthy, unrelated and genetically-diverse chicks for release in the wild in Bulgaria.

## Conclusion

Since 2011, in the WRBC in Stara Zagora, Bulgaria, there is a captive breeding group of the globally-endangered saker falcons. The pairs were formed, based on their known pedigree in order for their offspring to be as genetically diverse as possible. The progeny are being released in the wild in the country with the aim to restore the population of the species. Ten years later, in 2021 and 2022, the WRBC team had the opportunity to conduct genomic evaluation of blood samples obtained from 12 founding birds and undertake microsatellite analysis of 30 sakers from the programme. The genomic analysis indicated that two of the individuals in the WRBC breeding group may be kin-related and precautionary measures were taken to avoid breeding them. The rest of the results confirmed the prior information and, in addition, revealed unknown connections between some of the sakers with missing or incomplete pedigrees, indicating they can and should be used together for a better genetic management of these and other species bred in captivity, especially if performed in a timely manner. The development of a greater number of microsatellite loci for sakers and other large falcons through new genomic techniques will greatly enhance this process.

## Figures and Tables

**Table 1.** Breeding saker falcons in the WRBC for the period 2011-2022.

Season	Birds in the breeding programme	Formed pairs	Pairs with breeding behaviour	Pairs which laid eggs	Pairs which reared chicks
2011	4	2	2	0	0
2012	18	8	7	2	1
2013	21	10	8	3	3
2014	20	10	8	5	4
2015	24	10	9	5	5
2016	22	9	8	5	2
2017	22	11	9	5	5
2018	22	11	10	6	6
2019	21	10	9	5	4
2020	25	10	9	9	3
2021	29	12	12	12	8
2022	28	13	9	5	5

**Table 2.** Sample details of 12 saker individuals used as founders of the captive population of the WRBC.

Individual ID	Ring	Name
184	CZ151592	Frodo
319	6-98569	Arnold
320	0621	Hedy
0155	084544	Luna
0092	6-98611	Bilbo
1546	CZ126556	Lucia
1544	SKVG3601	Thomas
1543	CZ120480	Eurydice
1542	0286 /G13 11	Orpheus
0103	1153	Barir
1754	IBRUK77684W	Ariel
0149	А1300Е898	Romeo

**Table 3.** Sample details of the 30 saker individuals from the captive population of the WRBC. Eleven of them were the same from the previous sample (in bold).

Hatched	Ring	Name
**2014**	**IRBUK77684W**	**Ariel**
**2006**	**6-98569**	**Arnold**
2015	CZ151595	Arwen
2003	8201823783300W	Bandit
**N/A**	**1153**	**Barir**
**2015**	**6-98611**	**Bilbo**
2014	6-60051	Boryana
2015	6-60020	DJ
2013	CZ147598	Dobry
2019	6-60012	Dracarys
**2011**	**CZ120480**	**Eurydice**
**2015**	**CZ151592**	**Frodo**
2012	6-98519	Gogo
2013	6-98808	Grum
**2009**	**0621**	**Hedy**
2015	CZ151594	Lobelia
**2010**	**CZ126556**	**Lucia**
2016	CZ147613	Maul
**2010**	**ZG110286/130**	**Orpheus**
2009	Z288	Penda
2008	SK074503	Pizho
2010	6-98568	Plamena
2019	5-105915	Pluto
2019	5-105916	Rhaegal
**1994**	**А1300Е898**	**Romeo**
2020	6-98573	Shira
**2010**	**SKVG3601**	**Thomas**
2014	BG11CAAWRBC007	Thomas II
2010	6-98779	Vulna
2016	BG613C2016PLAM008	Willow

**Table 4.** Sequence quality for the 12 samples of saker falcon before and after trimming and filtering using the programme FASTP v.0.20.0 (https://github.com/opengene/fastp).

Individual ID	Raw reads	Size	Filtered reads	Filtered reads (%)	Duplication rate	Insert size peak
0092A	355,290,258	33.4 GB	355,160,964	3.64E-04	0.0152282	269
0103A	174,086,976	16.5 GB	174,024,596	3.58E-04	0.0038	269
0149A	208,120,172	19.8 GB	208,001,018	5.73E-04	0.0062	269
0155A	255,429,480	24.3 GB	255,306,192	4.83E-04	0.0118	269
1542A	174,721,138	16.1 GB	174,663,876	3.28E-04	0.0073	269
1543A	254,042,750	23.1 GB	253,942,168	3.96E-04	0.0155	269
1544A	185,516,958	17.1 GB	185,467,582	2.66E-04	0.0067	269
1546A	241,417,820	22.7 GB	241,318,934	4.10E-04	0.0072	269
1754A	164,998,882	15.7 GB	164,885,096	6.90E-04	0.0035	269
184A	241,030,674	22.9 GB	240,921,062	4.55E-04	0.0153	269
319A	237,942,550	22.3 GB	237,832,056	4.64E-04	0.0114	269
320A	278,550,182	26.2 GB	278,419,226	4.70E-04	0.0174	269
Total	2,771,147,840	260.1 GB	2,769,942,770			

**Table 5.** Mapping statistics obtained after aligning the sakers’ reads to the chromosome-level genome assembly of gyrfalcons. “Mapped reads” indicates the number and percentage of reads that are mapped to the genome; “Mapped paired-end” indicates the number and percentage of paired-end reads that properly mapped to the reference genome.

Individual ID	Total reads	Mapped reads	% Mapped reads	Mapped paired-end	% Mapped paired-end
0092A	355,635,683	354,311,958	99.63%	349,903,098	98.52%
0103A	174,292,918	173,545,178	99.57%	171,874,088	98.76%
0149A	208,354,172	207,173,630	99.43%	204,734,796	98.43%
0155A	255,673,112	254,723,148	99.63%	251,812,422	98.63%
1542A	174,935,440	174,149,259	99.55%	171,854,942	98.39%
1543A	254,349,330	253,383,348	99.62%	249,725,366	98.34%
1544A	185,748,451	185,000,530	99.60%	182,659,534	98.49%
1546A	241,724,700	240,849,092	99.64%	237,734,840	98.51%
1754A	165,204,993	164,557,856	99.61%	162,506,184	98.56%
184A	241,253,321	240,250,110	99.58%	237,704,994	98.67%
319A	238,206,967	237,297,258	99.62%	234,424,374	98.57%
320A	278,908,043	277,847,380	99.62%	274,189,792	98.48%
Average	231,190,594	230,257,396	99.59%	227,427,036	98.58%

**Table 6.** Microsatellite analysis showing the probability that the sampled WRBC saker falcons are part of a family/cluster. Prob (Inc.) gives the probability that all members of the cluster have r = 0.5. Prob (Exc.) indicates the probability that no further members belong to the corresponding category r = 0.5 or full sibling cluster.

	Prob (Inc.)	Prob (Exc.)	Member 1	Member 2	Member 3	Member 4	Member 5
1	1	0.5134	Barir				
2	0.9843	0.984	Ariel				
3	0.9969	0.5424	Pluto				
4	0.9844	0.0963	Orpheus				
5	0.7411	0.141	Thomas				
6	0.9328	0.9328	Bilbo	Willow	Shira	Thomas II	
7	1	0.9115	Dobry	Dracarys			
8	1	0.5252	Vulna	Penda	Plamena	Gogo	Arnold
9	1	0.0937	Grum				
10	0.9464	0.0953	Lucia	Boryana			
11	1	0.548	Pizho	DJ			
12	1	0.8856	Lobelia	Frodo	Eurydice	Arwen	
13	1	0.1665	Hedy				
14	1	0.1037	Bandit				
15	1	0.0475	Rhaegal				
16	1	0.1086	Maul				
17	1	0.2344	Romeo				
